# Ileosigmoid fistula and delayed ileal obstruction secondary to blunt abdominal trauma: a case report

**DOI:** 10.1186/1752-1947-5-507

**Published:** 2011-10-05

**Authors:** Konstantinos Bouliaris, Dimos Karangelis, Konstantinos Spanos, Stylianos Germanos, Evangelos Alexiou, Anargyros Giaglaras

**Affiliations:** 1 General Surgery Department, General Hospital of Larissa, Greece; 2 Department of General Surgery, 404 Military Hospital Larissa, Greece; 3 Radiological Department, General Hospital of Larissa, Greece

## Abstract

**Introduction:**

Abdominal trauma is a source of significant mortality and morbidity. Bowel injury as a result of blunt abdominal trauma is usually evident within hours or days of the accident.

**Case presentation:**

A 38-year-old Caucasian Greek man presented with a subtle and delayed small bowel obstruction caused by a post-traumatic ileosigmoid fistula and ileal stricture four months after a road traffic accident.

**Conclusion:**

Delayed occurrence of post-traumatic small bowel stricture and ileosigmoid fistula is an uncommon surgical emergency. General surgeons as well as emergency physicians should bear this manifestation in mind should a patient return to the hospital several weeks or even years after blunt abdominal trauma with symptoms or signs of bowel obstruction.

## Introduction

The abdomen is the third most commonly injured body part following trauma [1]. In 85% of cases it is the result of blunt trauma [2,3]. Solid organs, such as the liver and spleen, are the most frequently injured; injuries to the bowel or mesentery are rare. Although small bowel injury has been reported to be the third most common injury in blunt abdominal trauma (BAT), it was diagnosed in only 1.1% of admissions after blunt injury. Only 0.3% of patients had a small bowel perforation in a multi-institutional study [4]. In the absence of shock and peritonitis, patients with BAT may be treated conservatively and observed with computerized tomography (CT) [5]. However, on rare occasions such patients can present later on with symptoms and signs of small bowel obstruction or perforation [6-13]. We present a case of ileal stricture and an ileosigmoid fistula as a result of BAT.

## Case presentation

A 38-year-old Caucasian Greek man presented to our emergency department complaining of a three-month history of intermittent abdominal pain and frequent episodes of diarrhea. He had a history of a previous admission in another surgical department four months earlier for BAT after a road traffic accident. At that time he underwent an abdominal ultrasound, which showed no intraperitoneal fluid or solid organ injury, and he was admitted for observation. We also recovered from his discharge note that, during the first 48 hours of his hospitalization, a progressive decrease in the hematocrit value from 41% to 28% was noted. An abdominal CT scan at that time showed a small amount of fluid in the rectovesical pouch with no solid organ abnormalities and a large hematoma in the subcutaneous fat tissue on both lumbar areas. He was hemodynamically stable and he had a transfusion with one unit of packed red blood cells and three units of fresh frozen plasma. He improved rapidly with conservative treatment and was discharged on the fifth day, asymptomatic. One month later he started to have episodes of vague abdominal pains and frequent episodes of diarrhea after meals. Our patient also mentioned that during that four-month period he had lost 10 kg in weight. Due to his fear of the resulting diarrhea, he had cut down on eating.

**Figure 1:**
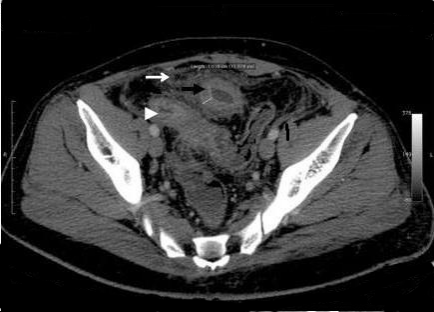
**Preoperative abdominal CT scan**. Shows a small bowel loop with a thickened wall and narrow lumen with proximal bowel dilation (black arrow). Increased density of the adjacent mesenteric fat (white arrow), as well the normal distal ileum (head arrow) can also be seen.

**Figure 2:**
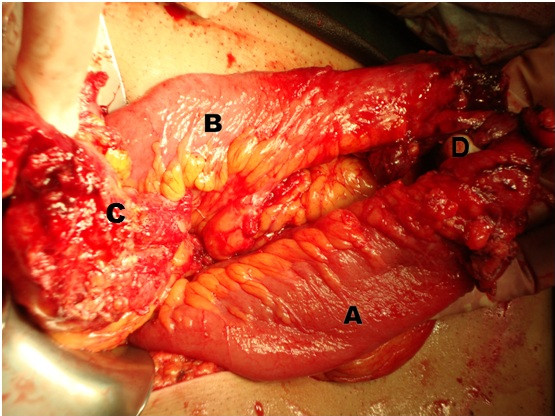
**Intraoperative image**. (A) Thickened proximal ileum; (B) normal distal ileum; (C) fistula point in sigmoid; (D) strictured ileum segment.

On our patient's current admission he complained of a colicky pain at the periumbilical region for the last 24 hours and two episodes of vomiting. On physical examination his abdomen was mildly distended with a diffuse tenderness on the hypogastrium. There was no rebound or guarding and palpation did not reveal any abdominal masses. His bowel sounds were increased. His blood tests were unremarkable. Plain abdominal X-rays revealed a dilated small bowel loop consistent with intestinal obstruction. He was initially treated with intravenous fluid replacement and nasogastric tube but his symptoms did not resolved. An enhanced-abdominal CT scan showed a small bowel loop with a thickened wall and narrow lumen with proximal bowel dilation. There was also increased density of the adjacent mesenteric fat (Figure 1). Bearing in mind the episodes of diarrhea as well as the loss of weight, we included Crohn's disease in our differential diagnosis. Further investigation with colonoscopy and a barium enema did not reveal any pathology. In view of his continuing symptoms and the radiological evidence of a small bowel obstruction, a laparoscopic exploration was carried out. Due to multiple adhesions though, we had to convert our plan to a laparotomy. During the operation we found a thickened segment of ileum in his pelvis adherent to his bladder and the apex of the sigmoid loop. There was a stricture in his ileum at this point, and an ileosigmoid fistula was present (Figure 2). The abnormal ileal loop was mobilized from his bladder and the sigmoid and resected, with restoration of intestinal continuity by primary side-to-side ileoileal anastomosis. The sigmoid fistula point was closed with seromucosal sutures. Histological examination of the resected specimen showed a mixed acute and chronic inflammatory process with hypertrophy of the muscularis externa. There was no evidence of Crohn's disease or malignancy. Our patient had an uneventful recovery and was discharged from hospital nine days later. At follow-up eight months later, he was symptom free and had regained weight.

## Discussion

Traumatic small bowel stricture and delayed small bowel obstruction secondary to BAT is a rare clinical entity. It is the result of local ischemia of the bowel wall and its subsequent healing with fibrosis and stricture, which causes the delayed onset of symptoms [12]. This local bowel ischemia can be caused by an injury to the mesentery, which impairs the blood supply to the bowel resulting in a stenotic segment, or a trauma which causes sufficient damage to the small bowel to result in hemorrhagic mucosal infarction or subclinical bowel perforation [8,9,12,13].

Patients suffering from post-traumatic small bowel obstruction usually present with intermittent abdominal pain and vomiting [9]. The interval between trauma and the onset of symptoms ranges from 13 days to 18 years, although the majority of patients experience symptoms within four to eight weeks of the initial trauma [9]. When small bowel obstruction is suspected, the investigation of choice is a small bowel contrast study or a contrast-enhanced CT [9,11,14]. Laparotomy and resection of the stenosed segment with primary anastomosis is the treatment of choice [9].

The presence of free intraperitoneal fluid in the abdomen without any evidence of solid organ injury is indeed an intriguing diagnostic challenge. Concerning the role of CT, it is unclear if it can provide a solid and conclusive answer as to whether surgery or close observation is best. Although CT exhibits very high sensitivity and specificity in detecting most solid organ injuries, it can still miss up to 15% of small bowel and mesenteric injuries [15-17]. We believe that laparotomy is not warranted in stable patients with free intra-abdominal fluid as a sole finding, who are otherwise fit and cooperative. We suggest close monitoring of these patients, including continued physical examination along with further testing if there is any doubt. We would consider surgical intervention as the last resource in our medical quiver, and only in a deteriorating patient. Many other authors agree that a trace of free fluid in these patients (given no other signs of injury) is not associated with significant intra-abdominal injury and can be safely managed nonoperatively [18,19]. Our case supports the subclinical perforation theory, with the perforation probably sealed by the adjacent sigmoid which led to the ileosigmoid fistula formation. This ileosigmoid fistula was the cause of the diarrheic episodes our patient experienced when the intraluminal pressure in the strictured ileum was raised. To the best of our knowledge, this combination of delayed ileum stricture and ileosigmoid fistula formation after BAT has not previously been described in the literature. The main differential diagnosis in these patients should be made from Crohn's disease [9-11,20].

## Conclusion

Patients with BAT who have small amounts of intraperitoneal fluid as the only finding on CT and are hemodynamically stable can be safely managed without surgical intervention. Delayed occurrence of post-traumatic small bowel stricture and ileosigmoid fistula is indeed a rare entity. Diagnosis of post-traumatic small bowel stricture could be difficult but general surgeons and emergency physicians should bear in mind this clinical manifestation and remain vigilant, especially when a patient presents with free intraperitoneal fluid after BAT on imaging, even if there are no signs of solid organ injury.

## Consent

Written informed consent was obtained from the patient for publication of this case report and any accompanying images. A copy of the written consent is available for review by the Editor-in-Chief of this journal.

## Competing interests

The authors declare that they have no competing interests.

## Authors' contributions

BK performed the literature search and was the chief author in writing the manuscript. DK performed the literature research and co-authored the paper. KS was the attending surgeon of the case and checked the paper. SG assisted with the linguistics and performed the literature research. EA helped with illustrations and submitted the radiological images. GA was the chief surgeon and performed the final check of the paper. All authors read and approved the final manuscript.
